# Promoting Physical Activity With Self-Tracking and Mobile-Based Coaching for Cardiac Surgery Patients During the Discharge–Rehabilitation Gap: Protocol for a Randomized Controlled Trial

**DOI:** 10.2196/16737

**Published:** 2020-08-19

**Authors:** Chao Zhang, Mohamed Soliman-Hamad, Roxanne Robijns, Niels Verberkmoes, Frank Verstappen, Wijnand A IJsselsteijn

**Affiliations:** 1 Eindhoven University of Technology Eindhoven Netherlands; 2 Catharina Ziekenhuis Eindhoven Eindhoven Netherlands; 3 Scamander Utrecht Netherlands

**Keywords:** self-tracking, mobile-based coaching, cardiac rehabilitation, randomized controlled trial, mHealth, eHealth

## Abstract

**Background:**

Home-based cardiac rehabilitations (CRs) with digital technologies have been researched and implemented to replace, augment, and complement traditional center-based CR in recent years with considerable success. One problem that technology-enhanced home-based CR can potentially address is the gap between cardiac interventions and formal CR programs. In the Netherlands and some other countries (eg, Australia), patients after cardiac interventions stay at home for 3-4 weeks without much support from their physicians, and often engage in very little physical activity (PA). A home-based exercise program enabled by digital technologies may help patients to better prepare for the later center-based CR programs, potentially increasing the uptake rate of those programs.

**Objective:**

In a randomized controlled trial (RCT), we will evaluate the effectiveness of a home-based walking exercise program enhanced by self-tracking and mobile-based coaching (treatment condition), comparing it with a version of the same program without these technologies (control condition). The added value of the digital technologies is justified if patients in the treatment group walk more steps on average (primary outcome) and show better physical fitness in a bicycle ergometer test and higher self-efficacy toward PA (secondary outcomes).

**Methods:**

Based on a power analysis, we will recruit 100 cardiac patients and assign them evenly to the 2 parallel groups. Eligible patients are those who are scheduled in the postanesthesia care unit, know the Dutch language, have basic literacy of using smartphones, and are without medical conditions that may increase risks associated with PA. In a face-to-face meeting with a nurse practitioner, all patients are prescribed a 3-week exercise program at home (2 walking exercises per day with increasing duration), based on national and international guidelines and tailored to their physical conditions after cardiac intervention. Their physical activities (daily steps) will be measured by the Axivity AX3 accelerometer worn at hip position. Patients in the treatment group will also be supported by a Neo Health One self-tracking device and a mobile platform called Heart Angel, through which they are monitored and coached by their nurses. After the study, all patients will perform a bicycle ergometer test and return the devices within 1 week. In addition, 5 questionnaires will be sent to the patients by emails to assess their self-efficacy toward PA and other psychological states for exploratory analyses (at discharge, at the end of each monitoring week, and 1 week after the study). To minimize bias, the randomization procedure will be performed after introducing the exercise program, so the nurse practitioners are blind to the experimental conditions until that point.

**Results:**

The study protocol has been approved by the Medical Research Ethics Committees United on February 26, 2018 (NL 62142.100.17/R17.51). By the end of 2018, we completed a small pilot study with 8 patients and the results based on interviews and app usage data suggest that a larger clinical trial with the targeted population is feasible. We expect to complete the RCT by the end of 2021, and statistical analyses will follow.

**Conclusions:**

Results of the RCT will help us to test the hypothesized benefits of self-tracking and mobile-based coaching for cardiac patients in home-based exercise programs during the discharge–rehabilitation gap. If the results are positive, cost-effectiveness analysis will be performed based on the insights of the study to inform the translation of the technology-enhanced program to clinical practice. We also note limitations of the trial in the discussion.

**Trial Registration:**

Registered at Netherlands Trial Register NL8040; https://www.trialregister.nl/trial/8040

**International Registered Report Identifier (IRRID):**

PRR1-10.2196/16737

## Introduction

Cardiac rehabilitation (CR) is an evidence-based and widely recommended strategy for the secondary prevention of cardiovascular diseases. Recent reviews have shown that CR is efficacious and cost-effective not only for reducing mortality by up to 26%, but also for preventing hospital re-admissions and improving quality of life [1-4]. In the Netherlands, after cardiac intervention, such as coronary artery bypass graft surgery, it is a standard practice to refer patients to participate in outpatient rehabilitation programs for 6-12 weeks, where group-based trainings of physical activity (PA), nutrition, and other lifestyle aspects are applied [5]. However, despite the proven benefits and the enforced practice, a general challenge for CR worldwide is the low rate of participation and completion by patients [5-8]. According to a Dutch cohort study [5], although patients after cardiac surgeries had the highest uptake rate among all cardiac patients, the number was just above 50%.

A particular challenge in the Netherlands is that there is usually a gap of 3-4 weeks between the discharge after cardiac surgery and the start of the formal CR, during which patients stay at home without much guidance on what they can do to improve their fitness. The same issue has also been reported in Australia [6]. The lack of guidance, combined with their low levels of self-efficacy for PA after surgery [9] and sometimes their preceding sedentary lifestyle, results in postsurgery PA levels that are below the level needed for optimal recovery [10]. Therefore, there is clearly a missed opportunity in this gap, which could be used to better prepare patients for the formal CR programs and even to increase the uptake rate of the programs. Moreover, because people are generally more susceptible to behavior change right after disruptive life events (eg, a cardiac surgery) [11], training patients at home for regular PA may also foster a long-term active lifestyle. To realize this opportunity, a home-based solution for supporting PA is needed.

In recent years, home-based CR has been gradually accepted as an alternative to the more traditional center-based CR. Evidence has been accumulated that home-based CR programs are often as effective as center-based CR programs [12-15], and they may incur lower costs [16,17] (but see [18]). A practical barrier for traditional CR is also eliminated as patients can stay at home and contact doctors or trainers through information and communication technologies (ICTs) only when necessary. Although long-term effects of home-based CR on PA and fitness are still in question [16], for our goal of preparing patients for the formal CR, a home-based approach is well suited in theory. However, most of the studies on applying ICTs in CR have focused on either replacing traditional CR with home-based CR [16,19] or augmenting ongoing center-based CR with new technologies [20,21], but much less on complementing the existing CR practice. One recent study examined the potential benefits of an internet- and mobile-based intervention on the maintenance of PA after regular CR, but the high attrition rate in the randomized controlled trial (RCT) prevented the authors from drawing clear conclusions [22]. More relevant to our research question, an ongoing RCT was designed to evaluate the use of SMS text messaging to support patients in the transition period after acute coronary events and before the start of CR [6]. More research is clearly needed to understand whether home-based interventions with ICTs before or after center-based CR are beneficial to patients.

Designing a cost-effective home-based PA intervention also raises the question of what intervention components are essential for an intervention to be efficacious. One component emphasized and utilized in almost all CR programs is personalized guidance from physicians based on patient monitoring [16], which can be implemented through several different communication modalities, including face-to-face meetings [23], phone calls [16], SMS text messages [24], and chat sessions using websites or mobile apps [19]. From a theoretical perspective, physician guidance plays multiple roles, such as goal setting, social persuasion, and emotional support, and it can even be more important for patients who have low cognitive capacities, or low self-efficacy toward PA after surgery [9,10]. Regardless of which modality to use, any physician guidance will increase workloads for health care professionals, so its benefits and costs are ought to be carefully weighted.

Another promising component is the self-monitoring of behavior and health status by patients themselves, usually supported by mobile and wearable self-tracking devices. Psychological theories generally consider self-monitoring as an important mechanism in self-regulation [25], and meta-analyses have demonstrated the effectiveness of self-monitoring as a behavior change technique [26]. Several RCTs examined the applications of self-tracking technologies in related clinical settings [27-29]. In one trial after myocardial infarction [27], patients who were required to record their weight, PA levels, blood pressure, and heart rate during a standard CR program reported higher self-efficacy toward PA and also exercised more than their peers in the control group 1 year later. In another trial [28], the use of pedometer alone led to higher levels of PA that were maintained 6 weeks and 6 months after a CR program. Finally, in [29], patients who wore an electronic step tracker and followed a home-based CR program showed similar levels of physical improvements to those who followed a center-based CR program.

In this study, we compare the effectiveness of 2 home-based PA programs that fill the gap between discharge and formal CR for patients after cardiac surgery, one with the support of digital technologies and one without this support. In the technology-enhanced group (intervention group), patients use a wristband to track their steps and communicate with physicians through a mobile app for 3 weeks. In the control group, patients are only told to follow a specific PA program at discharge, but do not receive any additional support while at home. Both trial conditions can be considered as clear improvements over the usual care, but they may differ greatly in their effectiveness and costs. We hypothesize that the use of self-tracking and mobile-based coaching will lead to higher PA levels and potentially also bring physical and psychological benefits at the end of the program, which would provide a rationale to implement these technologies into clinical practice. At the same time, costs associated with the technology-enhanced program can be estimated and used in cost-effectiveness analysis in the future.

## Methods

### Design

**Figure 1:**
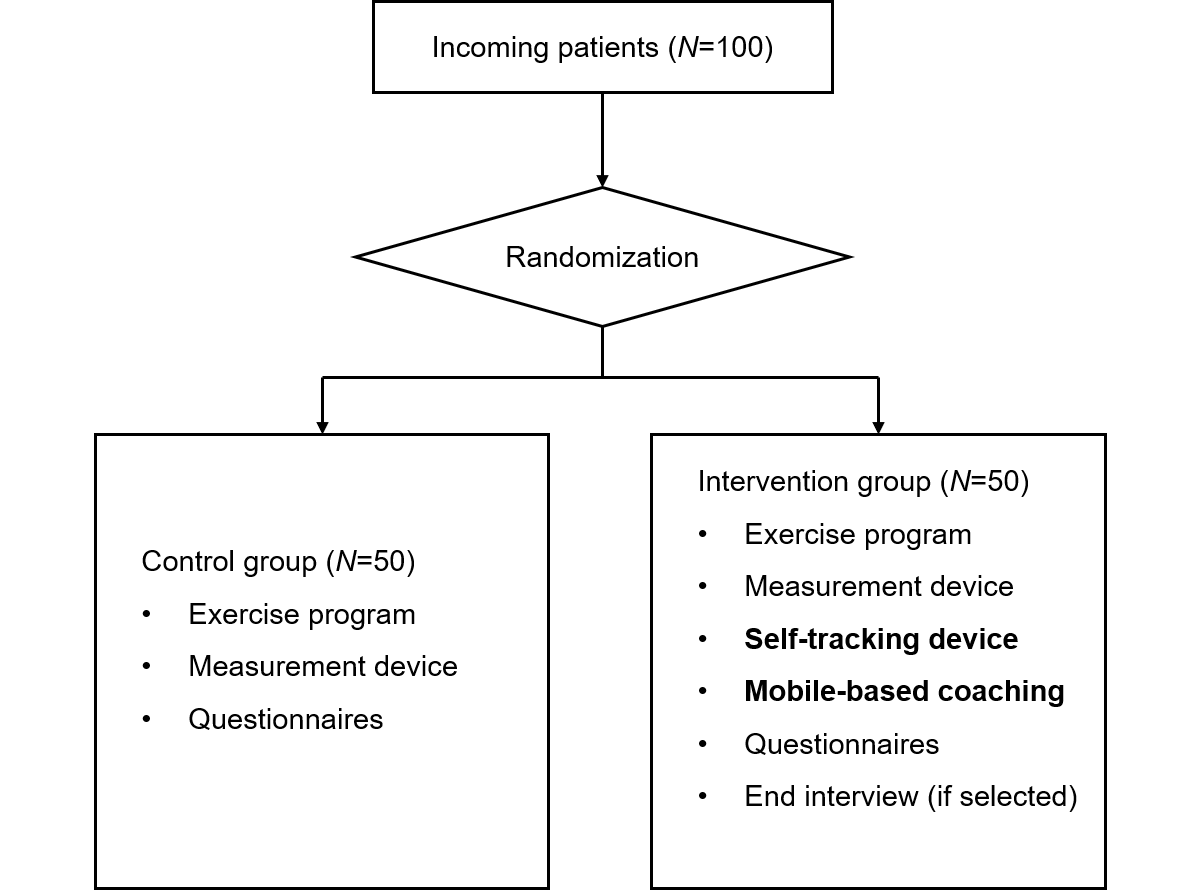
Design of the randomized controlled trial.

The study (registered at Netherlands Trial Register NL8040) will have a parallel-group randomized experimental design with an allocation ratio of 1:1. Participants will be randomized to the intervention or the control group during the introduction meeting at the date of discharge (Figure 1). Variables regarding participants’ psychological states (eg, self-efficacy toward PA) will be measured repeatedly over the weeks during the experiment (see the “Outcomes” section for details). The protocol conforms to the SPIRIT 2013 statement [30] and is described according to the CONSORT-EHEALTH checklist [31] (Multimedia Appendix 1).

### Eligibility and Recruitment

Patients who are expected to undertake a cardiac intervention at Catharina Ziekenhuis Eindhoven (CZE) and to follow a conventional CR program at least four weeks after discharge are eligible for this study. Eligible patients should also be scheduled as postanesthesia care unit patients, who are at relatively low risk and are expected to recover smoothly. Definition of postanesthesia care unit patients is well-operationalized in preoperative workup. Other inclusion criteria are (1) the patients are in possession of an Android or an iOS device (eg, a smartphone or a tablet), and have sufficient level of digital literary to use the device (eg, regular previous uses of emails and mobile apps); and (2) they understand the Dutch language. Patients are excluded if they are immobile, have a history of stroke with remaining dysfunction, or have serious complications, including stroke, severe myocardial infarction with echocardiography-confirmed myocardial function loss, or a serious infection that requires prolonged hospital stay and intravenous antibiotics.

Patient are always screened at the outpatient clinic at least three weeks before the operation. During the screening, information concerning the study are provided and the patient is given enough time (at least one week) to read the information and to ask questions. Signing the informed consent takes place during the admission 1 or 2 days before the operation.

### Sample Size Calculation

We used a minimum effect size of interest approach to calculate the sample size needed to detect a statistically significant difference between the intervention group and the control group in terms of the primary outcome (average daily steps over 3 weeks). In an internal discussion between researchers, physicians, and CR experts at CZE, a 2000-step daily difference was proposed to be the minimum effect size of interest, and a 3000-step within-group standard deviation was assumed based on [32]. These assumptions amount to a moderate to strong effect size in terms of Cohen *d* of 0.67, which is justifiable given the large differences between the two conditions in terms of costs. Based on a simulation study in the R statistical programming environment [33], 100 participants (50 in each group) are required to have a 90% power to detect the effect at a significance level of .05. Because we will also have day-level data for steps over 3 weeks, multilevel regression models can also be used for examining the differences in PA between the two groups, which should have much higher power due to the large number of observations (ie, data collected over 21 days by 100 patients). Details of the simulation-based power analysis (and the R code) can be found in Multimedia Appendix 2.

### Interventions

#### The Daily Exercise Program

**Figure 2:**
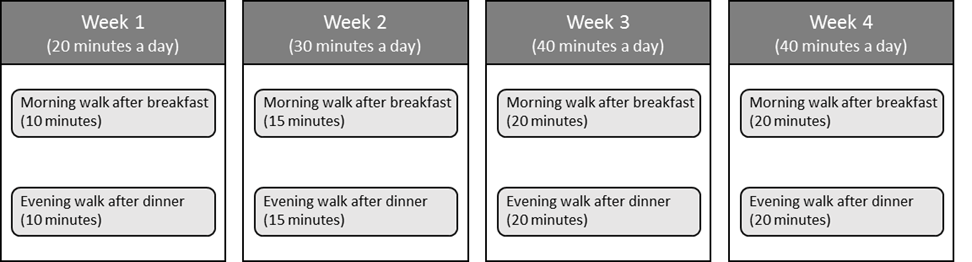
An example of a personalized rehabilitation plan for a patient.

The daily walking exercise program was designed based on both Dutch and European PA guidelines for the prevention of cardiovascular diseases and its rehabilitation [34,35], and also in compliance with expert opinions from physiotherapists at CZE. The program consists of 2 walking exercises and 1 overall goal for the amount of PA per day, with increasing intensity over the duration of the trial. The restricted durations of each walking exercise session are between 5 and 15 minutes for the first week, 10 and 20 minutes for the second week, and 15 and 25 minutes in the third week. The exact duration in each week will be decided by the patients and their physician together in the introduction meeting, adapted to the physical conditions of the patients after surgery (see Figure 2 for an example). Patients are also told to continue the same walking exercise after the 3-week study as long as the exercise plan does not conflict with their formal CR program.

One feature of the program is that patients will be encouraged to execute the 2 daily walking exercises at the same time and in the same context throughout the weeks. According to psychological theories of habit [36], performing the exercise in this way facilitates the formation of a strong exercise habit through the repetition of the behavior in the same environment. Based on the idea of implementation intention [37], patients will be asked to decide the time and context themselves according to their existing daily routines, and to imagine themselves performing the walking exercises in that context for 1 minute. This feature was used not only to prevent patients from omitting the exercise, but also to potentially promote a habit of exercising after the trial. The daily exercise program will be introduced to the intervention and the control group indifferently, and a booklet of the planned program will be distributed to all patients.

#### The Self-Tracking Device

**Figure 3:**
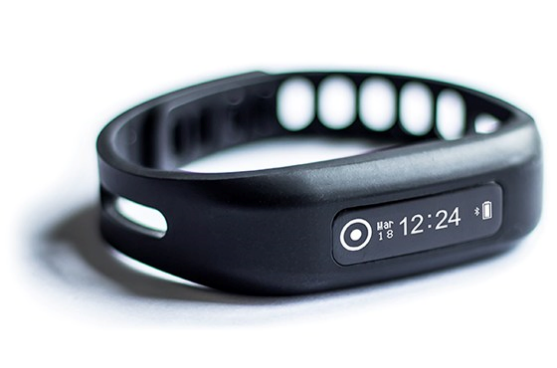
The Neo Health One wristband as the self-tracking device in the study.

Patients in the intervention group will receive a Neo Health One wristband (Figure 3), which is a commercial and CE-marked self-tracking device with build-in accelerometers. Patients are instructed to wear these bands as much as possible during the day. They can review at any time the number of steps they walked on a specific day and the step data are also synchronized to the mobile-based coaching app used in the trial. Patients in the intervention group will be instructed to wear the device as a wristband on their nondominant side.

#### The Mobile-Based Coaching App

Patients in the intervention group will also be monitored and coached by nurse practitioners through an e-coaching app called Heart Angel, which is a customized version of the commercial Virtuagym platform [38]. We decided to use this particular app because (1) the platform meets our needs for e-coaching and is free; (2) a customized version tailored to the context of CR was made available; and (3) the platform works seamlessly with the Neo Health One self-tracking device. The nurse practitioners work at the same cardiac surgery department at CZE and are known by the patients during their stays at the hospital, and they receive a 1-day training to use the platform provided by the researchers. The patients will use a mobile version of the Heart Angel app, while the nurse practitioners will use an associated web platform for coaching.

**Figure 4:**
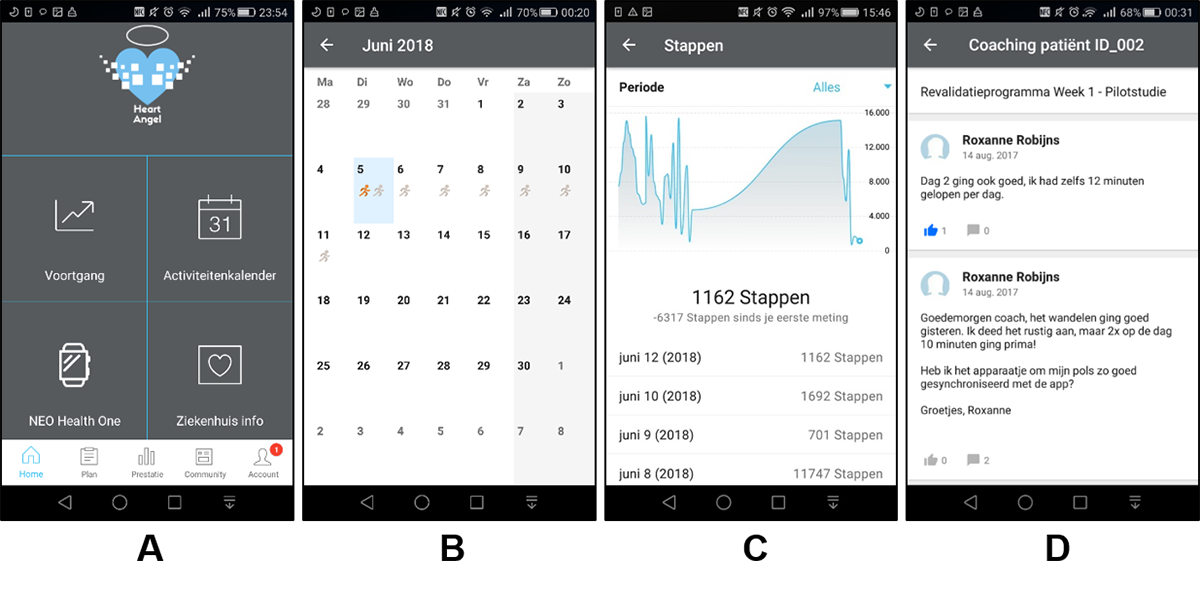
Screenshots of the Heart Angle mobile coaching app. (A) Homepage of the app showing the navigations to different functions. (B) Recorded walking exercise plan in the app in a calendar view. (C) Progress page showing the step data from the Neo Health One band. (D) An example of chatting between a patient and a coach.

The Heart Angel app serves the following functions. First, the daily walking exercise program is saved and shown in the app, so the patients can always open it to review their goals and plans (Figure 4B). Second, data from the self-tracking device are transmitted to the app, so both patients and their coaches (the nurse practitioners) can monitor their PA performance using the app (Figure 4C). Third, the mobile app sends daily notifications in the morning to remind the patients of their planned daily step goals, and whenever a goal is reached, they receive achievement badges from the app. Fourth, when there is a need to contact their coach, the patients can send messages through the mobile app (Figure 4D). Conversely, the nurse practitioners are asked to closely monitor their patients’ compliance and performance, and to coach them whenever needed through the app, for example, to check for problems if a patient is not exercising sufficiently according to the plan, or to compliment a patient who is doing well. As the above functionalities are prototypical for e-coaching platforms in general, our RCT is meant to be an evaluation of the general functionalities of e-coaching rather than the Heart Angel app per se.

### Primary and Secondary Outcomes

**Figure 5:**
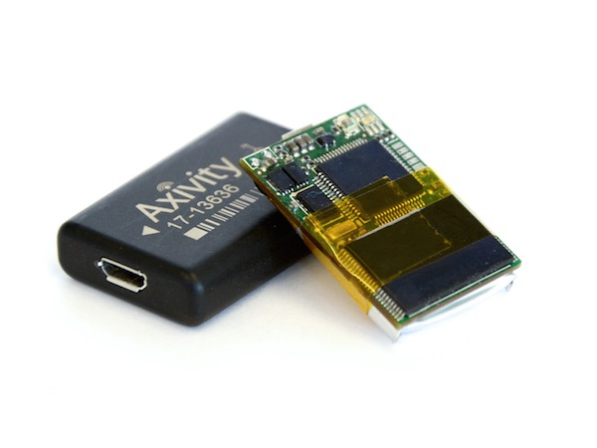
The Axivity AX3 accelerometer as the measurement device.

The primary outcome is the amount of daily physical activities by the patients, operationalized as the average daily steps over the 3 weeks. The steps are measured by the Axivity AX3 tracker, which is a CE-marked 3-axis logging accelerometer developed by the Open Movement organization at Newcastle University (Figure 5). The device is intended to be used in research on activity recognition, motion measurement, medical research, and movement science. It has been used previously in a large-scale clinical trial in UK [39] and validated for measuring PA in older adults [40]. Patients in both intervention and control group will be asked to wear the device as a clip on their cloths at their hip on the nondominant side (eg, a belt clip). The device has a long battery life (around 1 month) and large internal memory (1-month’s data with a sampling frequency of 50 Hz), so the patients do not have to worry about charging the device or offloading the data. Unlike the self-tracking device (the Neo Health One), this measurement device only records data but provides no feedback about steps to the patients.

In addition to the primary outcome, 2 secondary outcomes are used to test whether the behavioral-level increase of daily PA levels would bring patients benefits at physical and psychological levels for their preparations for the formal CR program. To measure physical fitness, a bicycle ergometer test will be held for each patient at the Cardiac Function Department as their usual clinical practice within 1 week after the 3-week trial. The research team will not be involved in the administration of the test, but 3 parameters of interest, namely, peak oxygen uptake (peak VO_2_), maximal workload, and maximum heart rate (HR_max_), will be provided to us. Previous studies have shown positive effects of walking trainings on physical fitness [41-43]. However, because of the short duration and moderate intensity of our exercise program, we do not strongly predict significant effects given the sample size.

Psychologically, we will examine whether patients in the intervention group report higher level of self-efficacy toward PA compared with the control group at the end of the third week. The rationale is that patients in the intervention group receive more guidance, social and emotional supports, and their ability to walk more steps may also increase perceived efficacy [44]. Based on a theory-based recommendation in [45], a scale for measuring self-efficacy in our context was developed specifically for this study and will be included in the 5 questionnaires sent to the patients (Multimedia Appendix 2).

### Additional Measurements for Exploratory Analyses

Besides primary and secondary outcomes and demographic and clinical data collected as part of the usual care, we will also collect app usage data and additional self-report measures for exploratory analyses. App usage data can provide preliminary insights into the role of specific features of the app in its overall effectiveness by correlating outcome variables with usage metrics, including login frequency, frequency of monitoring one’s step data (Figure 4C), coaching message frequency, and coaching message content.

Additional self-report measures are used to assess patients’ baseline PA levels before their cardiac events and to examine the dynamics of their psychological states over the weeks between the intervention and the control group. A complete list of the self-report measures in English can be found in Multimedia Appendix 2. All questions will be translated into Dutch for the trial.

#### Physical Activity Levels Before Cardiac Events

Patients’ regular PA levels before their cardiac events will be measured using the validated International Physical Activity Questionnaire [46], available in Dutch.

#### Trait Self-Control

Trait self-control has been shown to relate positively to a variety of health outcomes [47,48] and to people’s abilities to form healthy lifestyle habits [49]. However, the role of self-control in CR following cardiac intervention has not been examined. We will use the 10-item Brief Trait Self-Control Scale [50].

#### Attitude Toward the Daily Walking Exercise

Attitude refers to the subjective evaluation of a behavior object as positive or negative [51], and it is one of the most studied and important determinants of behavior change [52]. We will explore how patients’ attitude toward PA changes over time and whether self-tracking and mobile-based coaching lead to more positive attitude. Attitude is measured using seven 7-point semantic differential scales, for example, *good*–*bad*, *beneficial*–*harmful*, *pleasant*–*unpleasant*.

#### Habit Strength of the Daily Walking Exercise

As a patient repeats the daily walking exercise in a stable daily environment, the behavior will potentially become habitual and more likely to be maintained outside the context of the study [36]. We are interested in the dynamics of this habit formation [53] and how the intervention and control groups would differ in this regard. The validated 4-item Self-Report Behavioral Automaticity Index will be used [54].

### Randomization and Blinding

Patients will be allocated to the intervention or control group through a computerized randomization procedure during the introduction meeting. Specifically, the procedure takes place after the introduction of the general study information and the daily walking exercise program, and before the tutorials about how to use the (condition-dependent) technologies. The timing of the randomization is designed to blind the experimenter (a nurse practitioner) to treatment allocation until the daily walking exercise program is fully introduced, in order to minimize potential biases in guiding and motivating the patients. As with most electronic health (eHealth) trials, patients in our trial are not blinded to their conditions. Assessors of the bicycle ergometer test will be blinded to the research purposes as well as treatment allocation.

### Patient Timeline

**Figure 6:**
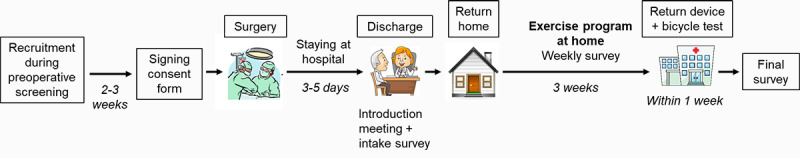
Patient timeline in this randomized controlled trial.

Patient timeline in this RCT is illustrated in Figure 6. During the preoperative screening in the outpatient clinic, patients are informed about the study and are given 1-2 weeks to make their participation decisions. If they agree to participate, a consent form is signed a few days before the surgery. After the surgery and the 3-5-day monitoring at the hospital, an introduction meeting with a nurse practitioner is held at discharge. In this meeting, the study goals and procedure are introduced first, and then the daily walking exercise program is explained and details are planned together with the patients. After the randomized group allocation, according to the assigned group, patients are handed the devices and are guided through the installation, registration, and use of the devices and the mobile app. After returning home, patients are expected to follow the exercise program at home for 3 weeks, before they are referred to the conventional CR program. Right after discharge and at the end of each study week, a questionnaire is sent to the patients by email to measure the variables of interest discussed above. After the 3-week trial, a bicycle ergometer test is scheduled to take place at the hospital within 1 week, where patients also return the devices. A final questionnaire is sent 1 week after the study to measure the same set of variables to explore the prolonged effects of the exercise program on patients’ psychological states. Eight patients from the intervention group will be randomly selected for an additional interview about their experience with the devices and the mobile app.

### Feasibility Study

To access the feasibility of the RCT (eg, whether patients after surgery can handle the devices), we conducted a small-scale trial with 8 patients in 2018. These patients followed the procedure for the intervention group in the RCT, except that the trial duration was only 1 week. Feasibility was assessed based on the usage data of the devices and the mobile app, a questionnaire that measures relevant psychological variables, and an interview (face-to-face or by phone call).

### Statistical Analysis

For the outcome variables of interest, that is, average daily steps, overall compliance rate, and physical fitness measured in the bicycle ergometer test (peak VO_2_, maximal workload, HR_max_), the following 2-step procedure will be used. In the first step, independent sample *t* tests (unpaired) will be used to compare the outcome variables between the two study conditions. Second, when potential confounding variables are identified post hoc, they will be added to multiple regression models to control for their influences. For step data at the day level, multilevel models will be built to test the same hypotheses, with patient ID as the grouping variable [55]. We will use the conventional α level of .05 to judge for statistical significance, and more importantly to use the estimated effect sizes in order to judge the clinical significance of the effects.

### Data Management and Ethics

The provider of the Heart Angel platform, Virtuagym, has a data management and privacy policy that is in accordance with the General Data Protection Regulation under the law of the European Union (GDPR) and Dutch laws for data protection. Because of the sensitive nature of the patient study, several additional measures will be taken to protect patients’ privacy. These include the following: (1) private chat groups will be created to only allow each patient to communicate with his/her coach but no others in a secured environment. (2) Questionnaire data will be stored on a local server, and no names or email addresses but only participant IDs will be used to identify questionnaire data. The participant IDs will not be used for the Axivity AX3 nor for the Neo Health One devices, in order to prevent risks of data breach in cases where the devices are lost. When these devices are returned, data will be removed from the devices and saved on a secured local computer at CZE. After the study, all data on the server of Heart Angel will be deleted, including patients’ user accounts, and only anonymous and aggregated data will be archived for research purposes.

Considering the low risk of this RCT, we have been granted an exemption from insurance by the Medical Research Ethics Committees United (MEC-U) according to Article 7, paragraph 6, of the Medical Scientific Research Act with People (WMO). The study protocol (including the feasibility trial) was formally approved by the MEC-U on February 26, 2018 (NL 62142.100.17/R17.51; see Multimedia Appendices 3 and 4), and by the local ethical committee at CZE in May 2018.

## Results

### Relevant Results From the Feasibility Study

**Figure 7:**
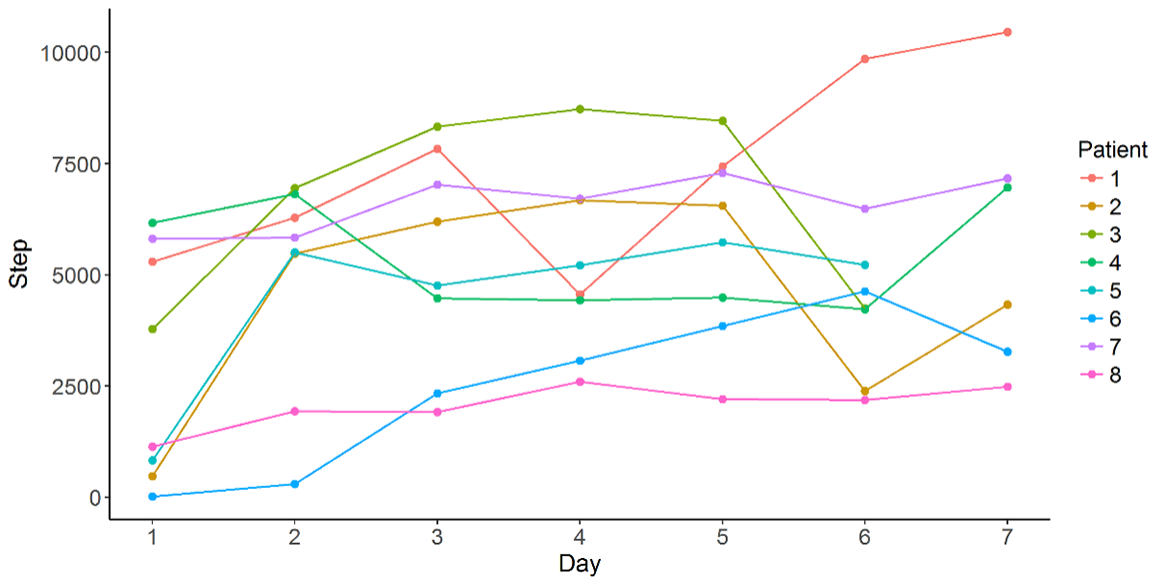
Number of steps walked for each patient throughout the 7-day feasibility study.

Results from the feasibility study strongly suggest that the planned RCT is feasible. First of all, from both interviews and objective data measured by the self-tracking device (see Figure 7 for all patients’ daily steps over the study week), it was evident that patients perceived the walking exercise very positively and they were able to almost always perform the exercise according to the plan. They also considered the exercise to be useful and pleasant to do, and did not experience any physical or mental problems with the exercise. A few patients even mentioned that they would like the durations of the exercise to be longer, and this proposition will be supported in the RCT with the increasing duration of the exercise over the 3 weeks.

Second, during the interviews, patients indicated that they had no trouble wearing both the Axivity AX3 and the Neo Health One devices. Sensor data from both devices suggest that the compliance rates of wearing the devices were close to 100% (54/56 days [96%] from 8 patients). When the patients went to sleep, they usually put the devices near their beds so that they will not forget wearing them in the next morning. Most patients found it convenient to wear one of the devices on the wrist and the other one as a clip on their belts or trousers. This observation and the fact that Axivity AX3 as a measurement device is more valid at lower limb positions [40] informed our decisions for the devices’ wearing positions in the protocol. In addition, all patients were able to fill out the questionnaire upon receiving emails.

**Figure 8:**
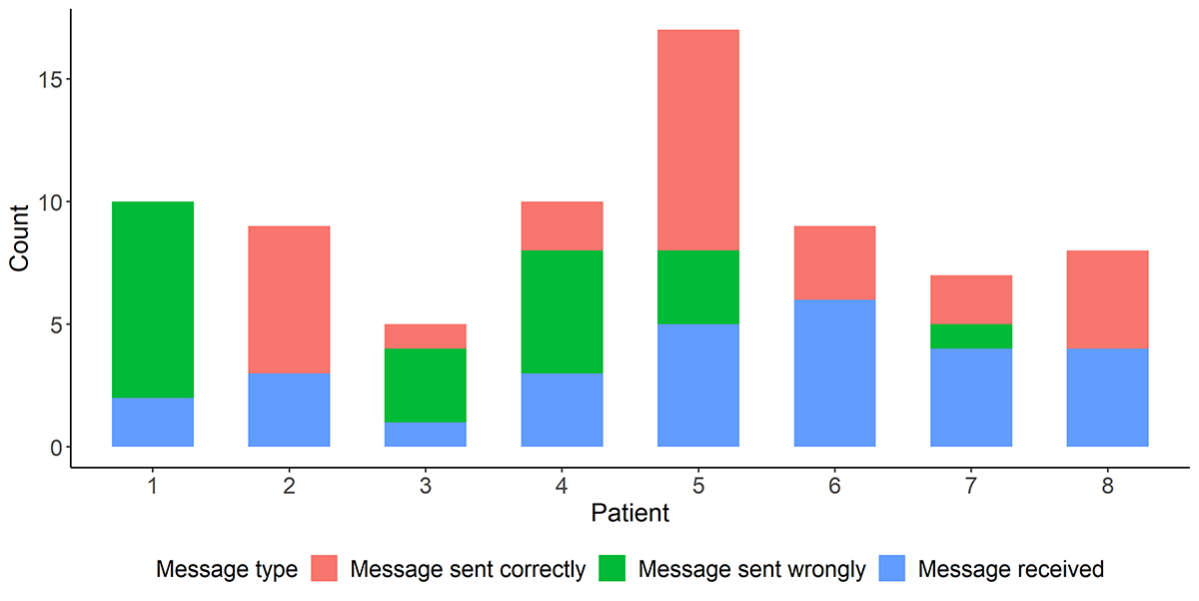
Number of messages sent to and from the patients.

Finally, usage data from the Heart Angel app showed that patients and coaches (the nurse practitioners) were able to use the app for chatting. Figure 8 shows the number of messages sent to and from the patients. We did find that some patients sent the messages to the wrong places—instead of sending directly to the coaches, the messages were posted to their public profile in the app. We will take this into account and explain the messaging function more clearly to the patients in the RCT.

### Expected Results for the Main RCT

We are planning to enroll the first patients in September 2020 and the RCT is expected to be completed by the end of 2021. After the completion, statistical analyses will be conducted based on the planned method reported in this protocol to determine if the two groups differ in the primary and secondary outcomes, in addition to the psychological variables under study.

## Discussion

We designed this RCT to evaluate the potential value of digital technologies—self-tracking and mobile-based coaching—in a home-based exercise program that fills the gap between surgery discharge and conventional CR programs. If our main hypothesis is confirmed (ie, patients in the intervention group would exercise more and become physically and psychologically better prepared for the formal CR than patients in the control group at the end of the trial), the study will provide a rationale to use self-tracking and mobile-based coaching in the home-based program. The technologies are also relatively cheap as, for example, the Heart Angel platform is free for users and a self-tracking device similar to Neo Health One could cost less than €20 (approximately US $22.5). Although a cost-effectiveness analysis is beyond the scope of this protocol, the results of the trial should provide good estimates about the benefits of the technologies and insights about the required workloads from nurse practitioners. If the hypothesis is not confirmed, the results may imply that giving patients a more structured exercise program in combination with an implementation intention procedure is sufficient to support them during the gap period without technologies.

One apparent limitation of the study is that the 2 home-based CR conditions are not directly compared with the usual care. Although ideally adding such a baseline condition is useful, given practical constrains (eg, time, financial resources) our design can be justified. Both previously published research [9] and expert opinions from local physicians suggest that under the current practice, patients are clearly not supported enough and they do not exercise enough during the gap period. Thus, compared with this *straw man* condition, the condition with the low-tech exercise program is a more meaningful baseline for evaluating the technologies, because it requires minimum effort to improve the usual care. Another limitation is that our experimental design does not allow us to disentangle the beneficial effects of self-tracking and e-coaching. For example, it might be that self-tracking is the main active component of the intervention, so the costs associated with human coaching can be saved. While future research is needed to accurately separate the effects, the planned exploratory analyses on app usage data may provide preliminary answers. A third limitation relates to potential reactivity to the measurement device in the control group. Although the Axivity AX3 device does not support self-monitoring, merely wearing the device may increase patients’ awareness of their PA levels and motivate to perform more activities [56-59]. This limitation does not invalidate the testing of the differences between the trial conditions, but caution should be exercised when generalizing the results of the control group to the low-tech solution in practice. Despite the limitations, our study should provide valuable data for bridging the discharge–rehabilitation gap in the current CR practice and more generally contribute to the growing literature on enhancing traditional center-based CR with digital technologies [6,21].

## Appendix

Multimedia Appendix 1 CONSORT-EHEALTH checklist.

Multimedia Appendix 2 Self-report measures.

Multimedia Appendix 3 Initial review and comments by MEC-U.

Multimedia Appendix 4 Second review and acceptance by MEC-U.

## Abbreviations

CR: cardiac rehabilitation
CZE: Catharina Ziekenhuis Eindhoven
GDPR: General Data Protection Regulation under the law of the European Union
HR_max_: maximum heart rate
ICT: information and communication technology
MEC-U: Medical Research Ethics Committees United
PA: physical activity
Peak VO_2_: peak oxygen uptake
RCT: randomized controlled trial
