# Supplementary material for: Promoting Physical Activity With Self-Tracking and Mobile-Based Coaching for Cardiac Surgery Patients During the Discharge–Rehabilitation Gap: Protocol for a Randomized Controlled Trial
Source: JMIR Res Protoc. 2020 Aug 19;9(8):e16737. doi: 10.2196/16737 (PMC7468644; doi:10.2196/16737)
Supplement: Multimedia Appendix 2 [file resprot_v9i8e16737_app2.docx]

**Appendix 2: Self-report measures**

**1a. Brief Trait Self-control Scale**

This will be measured only once after the introduction meeting.

*Please indicate how much the following statements reflect how you typically are (1 = Not at all; 7 = Very much)*

*1.1 I am good at resisting temptation.*

*1.2 I have a hard time breaking bad habits.*

*1.3 I am lazy.*

*1.4 I say inappropriate things.*

*1.5 I do certain things that are bad for me, if they are fun.*

*1.6 I refuse things that are bad for me.*

*1.7 I wish I had more self-discipline.*

*1.8 People would say that I have iron self-discipline.*

*1.9 Pleasure and fun sometimes keep me from getting work done.*

*1.10 I have trouble concentrating.*

*1.11 I am able to work effectively toward long-term goals.*

*1.12 Sometimes I cannot stop myself from doing something, even if I know it is wrong.*

*1.13 I often act without thinking through all the alternatives.*

**1b. International Physical Activity Questionnaire**

This will be measured only once, presumbly before the surgery.

<https://docs.google.com/viewer?a=v&pid=sites&srcid=ZGVmYXVsdGRvbWFpbnx0aGVpcGFxfGd4OjhlMTcxZGJkZmMxYTg1NQ> (English)

<https://docs.google.com/viewer?a=v&pid=sites&srcid=ZGVmYXVsdGRvbWFpbnx0aGVpcGFxfGd4OjJhNTgwN2Y1YTI1ZWE2MDM> (Dutch)

**2. Attitude and beliefs**

This will be measured after the introduction meeting and at the end of each study week (5 times).

*Doing the daily exercises in my rehabilitation program is...*

*2.1 Very bad 1 --- 2 --- 3 --- 4 --- 5 --- 6 --- 7 Very good*

*2.2 Very worthless 1 --- 2 --- 3 --- 4 --- 5 --- 6 --- 7 Very useful*

*2.3 Very harmful 1 --- 2 --- 3 --- 4 --- 5 --- 6 --- 7 Very beneficial*

*2.4 Very unpleasant 1 --- 2 --- 3 --- 4 --- 5 --- 6 --- 7 Very pleasant*

*2.5 Very foolish 1 --- 2 --- 3 --- 4 --- 5 --- 6 --- 7 Very wise*

*2.6 Very unhealthy 1 --- 2 --- 3 --- 4 --- 5 --- 6 --- 7 Very healthy*

*2.7 Very difficult 1 --- 2 --- 3 --- 4 --- 5 --- 6 --- 7 Very easy*

**3. Self-efficacy**

This will be measured after the introduction meeting and at the end of each study week (5 times).

*Please rate your confidence of doing the following walking exercises (0 = Not confident at all; 10 = Completely confident)*

*3.1 Walking continuously for 5 minutes*

*3.2 Walking continuously for 10 minutes*

*3.3 Walking continuously for 15 minutes*

*3.4 Walking continuously for 20 minutes*

*3.5 Walking continuously for 25 minutes*

*3.6 Walking continuously for 30 minutes*

**4. Behavioral automaticity**

This will be measured after the introduction meeting and at the end of each study week (5 times).

*Doing my afternoon walking exercise every day is...*

*4.1 ...* *something I do without having to consciously remember. (1 = Strongly disagree; 7 = Strongly agree)*

*4.2 ...* *something I do automatically. (1 = Strongly disagree; 7 = Strongly agree)*

*4.3 ...* *something I do without thinking. (1 = Strongly disagree; 7 = Strongly agree)*

*4.4 ...* *something I start doing before I realize I'm doing it. (1 = Strongly disagree; 7 = Strongly agree)*

*Doing my evening walking exercise every day is...*

*4.5 ...* *something I do without having to consciously remember. (1 = Strongly disagree; 7 = Strongly agree)*

*4.6 ...* *something I do automatically. (1 = Strongly disagree; 7 = Strongly agree)*

*4.7 ...* *something I do without thinking. (1 = Strongly disagree; 7 = Strongly agree)*

*4.8 ...* *something I start doing before I realize I'm doing it. (1 = Strongly disagree; 7 = Strongly agree)*

**5. Obstacles in rehabilitation program**

This will be measured after the preparation session and at the end of each study week (2 times in Study 1 and 5 times in Study 5)

*Please indicate whether you have experienced the following obstacles in your rehabilitation during the last week (1 = Not at all; 2 = A lot)*

*5.1 Worries about risks on my heart condition*

*5.2 Physical pains/uncomfort*

*5.3 Walking exercise not fit with my daily schedule*

*5.4 Concerns from family/friends*

*5.5. Cannot always remember*

*5.6 Lack of motivation*

*5.7 Lack of guidance and feedback from my coach*

*5.8 Other, please specify and rate _____________*
