# Supplementary material for: Promoting Physical Activity With Self-Tracking and Mobile-Based Coaching for Cardiac Surgery Patients During the Discharge–Rehabilitation Gap: Protocol for a Randomized Controlled Trial
Source: JMIR Res Protoc. 2020 Aug 19;9(8):e16737. doi: 10.2196/16737 (PMC7468644; doi:10.2196/16737)
Supplement: Multimedia Appendix 3 [file resprot_v9i8e16737_app3.pdf]

MEC-U
MEDICAL RESEARCH ETHICS COMMITTEES UNITED

Medical research Ethics Committees United

Postadres Postbus 2500
3430 EM Nieuwegein

Bezoekadressen Locatie Nieuwegein
Koekoekslaan 1 te Nieuwegein
030 609 3580

Locatie Eindhoven
Michelangelolaan 2 te Eindhoven
040 239 8607

E-mail info@mec-u.nl

Website www.mec-u.nl

Catharina Ziekenhuis
T.a.v. dr. M. A. Soliman Hamad, Research arts
Postbus 1350
5602 ZA EINDHOVEN

Kenmerk: V.113615/R17.051/dj/dj

Datum: 5-9-2017

Betreft: commentaar commissie NL62142.100.17
R17.051/E-coaching

Geachte heer Soliman Hamad,

Uw studie met titel: **"The Effectiveness of Self-monitoring and Web-based Coaching in Promoting Physical Activity during Early Cardiac Rehabilitation: A Feasibility Study & A Randomized Controlled Trial"**, registratienummer **R17.051** en acroniem **E-coaching**, is door de commissie besproken tijdens haar vergadering van 7 augustus 2017.

Uit deze vergadering zijn de volgende vragen en opmerkingen gekomen.

<u>Medisch/ethisch</u>

1. Uitkomstvariabelen van de gerandomiseerde studie worden in het onderzoeksprotocol niet duidelijk onder een apart hoofd samengevat. Deel staan ze onder punt 4.1.1. (procedure) en deels in appendix 3 (sample size estimate). Graag aanpassen.
2. Wanneer vindt de overgang van studie 1 naar studie 2 plaats?

<u>Methodologisch</u>

Naar de mening van de commissie is dit, uit de aard der zaak, geen dubbelblinde studie. Ten einde indicatie bias tegen te gaan wordt gerandomiseerd, dat is in een niet geblindeerde studie van eminent belang. Graag de randomisatieprocedure zo gedetailleerd omschrijven dat duidelijk wordt dat de uitkomst van een randomisatieprocedure niet te voorspellen is; vooralsnog voldoen alleen web-based randomisatie programma's; de enveloppen methode is onvoldoende. Als er na randomisatie verschillen blijken te bestaan van baseline karakteristieken, dan is dat door toeval ontstaan. De ernst van deze baseline verschillen is niet te kwantificeren met een p-waarde; het woord significant kan hier niet worden gebruikt in de statistische betekenis. Er blijft een probleem want alleen voor de gemeten baselinevariabelen kan worden gecorrigeerd in de statistische analyse. Aan te raden is om de primaire analyse een 'crude' analyse te laten zijn, d.w.z. een univariabele analyse (een Student t-toets b.v., omdat gesproken wordt van een gemiddelde en standaard deviatie) en pas in tweede instantie een multivariabele analyse toe te passen, want de extra variabelen in de multivariabele analyse worden post-hoc bekend. Om informatie bias tegen te gaan kan de meting van primaire uitkomstmaat blind gebeuren. Maar in dit geval is de uitkomstmaat redelijk hard, in de zin van niet te beïnvloeden door de onderzoekers. De sample size berekening gaat uit van de gewenste uitkomst. Indien wordt uitgegaan van de klassieke frequentistische statistische analyse hebben we hier te maken met een null-hypothese die zegt dat er geen verschil is tussen beide behandelstrategieën; alhier b.v. beide groepen gaan gemiddeld 5000 stappen halen met een s.d. van 3000. De alternatieve hypothese is dat

er wel verschil is tussen beide groepen. We willen de null-hypothese verwerpen als de kans < 5% dat een gevonden verschil door toeval is ontstaan; aangenomen een Student t-toets, een minimaal detecteerbaar verschil van 2000 stappen en een power van 90% geeft de formule (die hetzelfde gaat geven als uw iteratie, want u gaat daarbij uit van een normale verdeling en student t-toets) een aantal van 48 patienten in iedere groep.Een andere zienswijze op het minimaal detecteerbaar verschil is de verhouding verschil en standaard deviatie. Deze studie wil een verschil van 2/3 van de standaard deviatie meten, dat is zeer aanvaardbaar. De statistische analyse paragraaf dient wat uitgebreider te zijn, met expliciete vermelding van welke test a priori zal gaan worden gebruikt (kan worden onderverdeeld in primair en secundair).

<u>Productinformatie</u>
1. Fitbit; axivity AX3: CE conformity statement + intended use ontbreken. Graag indienen.
2. Self-tracking device (neo-health one): CE conformity statement + intended use ontbreken. Graag indienen.
3. Coaching website (Data angel), Hoe zit het voor dit product met databeveiliging? Communiceert patiënt op veilige manier met coach? Eis je bepaalde safety windows updates van patiënten thuis? Graag aantonen dat e.e.a. goed beveiligd is en geen gevoelige patiëntendata op straat kan komen. CE conformity statement + intended use ontbreken. Graag indienen.

<u>Juridisch</u>
1. Data storage: *'data Angel company may keep identifiable behavioural data of using the platform on its server'*. Dit is niet toegestaan. Graag wijzigen.
2. U heeft vrijstelling van verzekering gevraagd. De commissie kan dit op grond van artikel 7 lid 6 van de Wet medisch-wetenschappelijk onderzoek met mensen (WMO) verlenen indien het onderzoek tot doel heeft gebruikelijke handelingen te vergelijken en er hooguit verwaarloosbare risico's voor de proefpersonen verbonden zijn aan het onderzoek. De commissie is van mening dat aan deze twee criteria is voldaan, waardoor besloten is ontheffing te verlenen.

Ten aanzien van de documenten heeft de commissie de volgende vragen en opmerkingen:

Documenten specifiek

<u>B1. ABR-formulier, versie 01, d.d. 26-07-2017, niet getekend</u>
1. Vraag C17: graag het antwoord wijzigen in 'ja'.
2. Vraag C23a: graag de startdatum wijzigen.
3. Vraag D11: de commissie merkt op dat wanneer het interview / fitnesstest aan het einde van het onderzoek een extra ziekenhuisbezoek vereist, de reiskosten hiervoor aan de deelnemer dienen te worden vergoed. Graag aanpassen alsook de overige betreffende documenten.
4. Vraag E1: graag het antwoord wijzigen in 'ja'. Therapeutisch effect is verbetering conditie patiënt.
5. Vraag E4: antwoord is niet volledig; de fitness test, de interviews en het gebruik van het e-coaching system worden niet genoemd.
6. Vraag F1: niet duidelijk is hoe de patiënt wordt benaderd en of de onderzoeker die het contact legt in dit geval ook de behandelend arts is of een onafhankelijk persoon. Dit is van belang om te kunnen beoordelen of de werving op juiste wijze geschiedt. Graag nader toelichten. Zie ook de opmerking onder Onderzoeksprotocol.
7. Vraag G3: niet van toepassing. Graag aanpassen.
8. Sectie K: Nederlandse en Engelse versie wijken belangrijk af. Graag uniformeren.
9. De commissie wijst erop dat, alvorens goedkeuring te kunnen verlenen, zij dient te beschikken over een getekend exemplaar van het ABR-formulier.

<u>C1. Onderzoeksprotocol, versie 3, d.d. 01-06-2017</u>
1. Zie opmerkingen onder Methodologisch.
2. Graag intentie/mogelijkheid tot publicatie vermelden.
3. Graag informatie over ontheffing proefpersonenverzekering opnemen.
4. Graag het protocol nalopen op typefouten.

5. De hoofdonderzoeker genoemd in het onderzoeksprotocol (drs. Verberkmoes) komt niet overeen met de hoofdonderzoeker genoemd in het ABR-formulier en in de patiënteninformatiebrief (dr Soliman Hamad). Graag uniformeren.
6. In onderzoeksprotocol staat dat patiënten gevraagd worden om mee te doen voor de studie vlak na de operatie. Kan patiënt dan goed beoordelen of hij/zij mee wil doen? Dit matcht niet met patiënteninformatiebrief. Hierin staat dat de patiënt gevraagd wordt voor de hartoperatie. Hoe verloopt de werving van patiënten voor deelname aan dit onderzoek nu?
7. Wat bepaald overgang tussen deel 1 (haalbaarheidsstudie) en deel 2? Staat onvoldoende beschreven in onderzoeksprotocol.
8. "We will randomly select some participants from the intervention for a similar interview as in Study 1." Hoeveel is 'some'?
9. Gebruik van patiënten ID om data van diverse systemen achteraf te kunnen linken vindt de commissie gevaarlijk in verband met mogelijk verlies van devices gedurende de studie en daarmee een datalek.

<u>E1/E2. Patiënteninformatiebrief, incl. toestemmingsverklaring versie 1.1., fase 1, d.d. 04-05-2017 **en** fase 2 d.d. 12-05-2017</u>

1. Graag het ToetsingOnline nummer vermelden.
2. Graag het woord 'dokter' vervangen door 'arts'.
3. Het is niet duidelijk of patiënten mobiele telefoon met daarop de app ook altijd bij zich moeten dragen. Graag nader toelichten.
4. In onderzoeksprotocol staat beschreven dat meters af mogen tijdens slapen. Dit staat niet in patiëntenbrief. Mogen de meters nat worden? Douchen/bad?
5. In brief staat niet omschreven dat data ingezien wordt op TU/e (naast CZE). Graag aanpassen.
6. Zelfregulerende activiteitenmeter is moeilijk voor patiënten. Wat wordt bedoeld met zelfregulerend?
7. De paragraaf over vertrouwelijkheid/gebruik gegevens is te summier. Graag aansluiten bij DCRF-template te raadplegen via http://www.ccmo.nl/attachments/files/model-pif-nl-update-maart-2017.docx.
8. In de brief ontbreekt een stukje over de ontheffing van de proefpersonenverzekering. Graag toevoegen.
9. Aan het einde van de brief graag 'Dank voor uw aandacht' toevoegen.

<u>E1/E2. Patiënteninformatiebrief incl. toestemmingsformulier versie 1.1., fase 1, d.d. 04-05-2017</u>

1. Inleiding: graag aanpassen: '*Lees ook de brochure 'Medisch-wetenschappelijk onderzoek. Algemene informatie voor de proefpersoon'*. Graag de zin *'Daarin staat veel algemene informatie over medisch-wetenschappelijk onderzoek'* verwijderen.
2. Paragraaf 'Achtergrond & doel van het onderzoek': het ontwikkelen en testen *wat* vanuit huis gedaan kan worden. Graag aanpassen.
3. Paragraaf 'Achtergrond & doel van het onderzoek': graag een punt plaatsen achter online platform.
4. Paragraaf 'Hoe wordt het onderzoek uitgevoerd?': het zinsdeel *'voor de volgende vier weken dat u thuis zult zijn'*: graag laatste woorden wijzigen in *'dat u thuis bent'*.
5. Paragraaf 'Hoe wordt het onderzoek uitgevoerd?': 'U zal een brochure meenemen' graag wijzigen in 'U krijgt een brochure'.
6. Paragraaf 'Hoe wordt het onderzoek uitgevoerd?': Aan het einde van de week *'zult u bezoeken'* graag wijzigen in *'bezoekt u'* en *'een kort interview te houden'* graag wijzigen in *'krijgt u een interview'.*
7. Paragraaf 'Zijn er risico's verbonden bij deelname aan deze studie?: *'Na de operatie wordt uw persoonlijke staat nagekeken door een dokter (…)'* graag wijzigen in *'Na de operatie wordt u zorgvuldig nagekeken door een arts (…)'*.
8. Paragraaf 'Wat gebeurt er als u niet wenst deel te nemen aan dit onderzoek?': *'(…) kunt u zich altijd bedenken en toch stoppen. Ook tijdens het onderzoek'.* Graag toevoegen: *'U hoeft niet te zeggen waarom. Wel moet u dit direct melden aan de onderzoeker'.* Zie paragraaf 8 DCRF-template.
9. Toestemmingsformulier: graag wijzigen in 'Toestemmingsformulier proefpersoon'.
10. Toestemmingsformulier: graag eerste bullet verwijderen 'voor de patiënt (proefpersoon).
11. Toestemmingsformulier: zesde bullet graag veranderen in *'ik wil meedoen met dit onderzoek'*.

E1/E2. Patiënteninformatiebrief, incl. toestemmingsverklaring versie 1.1., fase 2 d.d. 12-05-2017

1. Inleiding: zie opmerking brief fase 1.
2. Paragraaf ‘Achtergrond & doel van het onderzoek’: *‘het ontwikkelen en testen dat vanuit huis uitgevoerd kan worden’* graag wijzigen in *‘wat vanuit de thuissituatie gedaan kan worden’* of *‘wat mogelijk is’*.
3. Paragraaf ‘Achtergrond en doel van het onderzoek’: *‘Daarnaast is er 50% kans dat u extra hulp zult krijgen met behulp van een zelfregulerende activiteitenmeter die (…)’*. Graag aanpassen.
4. Paragraaf ‘Achtergrond en doel van het onderzoek’: laatste zin graag wijzigen in *‘Er doen 100 patiënten mee aan dit onderzoek’*.
5. Paragraaf ‘Wat zijn mogelijke voor- en nadelen van deelname aan dit onderzoek?’: ‘Er is daarom een toegevoegde waarde voor uzelf als u deelneemt aan het onderzoek’. Graag deze zin verwijderen (te wervend).
6. Paragraaf ‘Zijn er risico’s verbonden bij deelname aan deze studie?: zie opmerking brief fase 1.
7. Paragraaf ‘Wat gebeurt er als u niet wenst deel te nemen aan dit onderzoek?’: zie opmerking brief fase 1.
8. Toestemmingsformulier: zie opmerkingen brief fase 1.

Cv’s

Graag een recent cv van hoofdonderzoeker, drs. N.J.E. Verberkmoes, indienen.

Overig

1. De vragenlijsten zijn nog in het Engels. Graag naar het Nederlands vertalen.
2. De commissie is van mening dat de huisarts geïnformeerd dient te worden. Graag een huisartsenbrief indienen. Graag informatie hierover toevoegen aan het protocol, de patiënteninformatiebrief en het toestemmingsformulier.

Conclusie

De commissie ontvangt graag antwoord op de gestelde vragen en de aangepaste documenten alvorens zij een beslissing neemt over de deugdelijkheid van het protocol. Tot die tijd wordt de toetsingstermijn opgeschort.

Bovenstaand commentaar is onder voorbehoud van de formele vaststelling van de notulen door de commissie tijdens de eerstvolgende vergadering.

Ter bevordering van een vlotte afwerking wordt u vriendelijk verzocht om:

- de vragen van de commissie puntsgewijs te beantwoorden;
- de aangepaste documenten te voorzien van een nieuwe datum en versienummer;
- de gewijzigde tekst in de documenten cursief weer te geven en weglatingen door te strepen;
- één "opgeschoond" exemplaar van de gewijzigde documenten bij te voegen.

Bij wijzigingen in het ABR-formulier dient de aangepaste versie opnieuw ondertekend te worden.

De commissie ontvangt uw antwoord graag binnen twee maanden na dagtekening van deze brief (antwoordbrief op papier en ondertekend en bijlagen, voorzien van benodigde handtekeningen, digitaal op CD-Rom of USB-stick). De beoordelingstermijn wordt opgeschort tot het moment dat wij uw reactie volledig hebben ontvangen.

Ik verwacht u hiermee voldoende te hebben geïnformeerd.

Met vriendelijke groet,

mw. mr. D.J. Joosten, ambtelijk secretaris

Namens dr. B. van Ramshorst
voorzitter Medical research Ethics Committees United (MEC-U)

C.c. drs. N.J. Verberkmoes onderzoeker Catharina Ziekenhuis
