# Supplementary material for: Promoting Physical Activity With Self-Tracking and Mobile-Based Coaching for Cardiac Surgery Patients During the Discharge–Rehabilitation Gap: Protocol for a Randomized Controlled Trial
Source: JMIR Res Protoc. 2020 Aug 19;9(8):e16737. doi: 10.2196/16737 (PMC7468644; doi:10.2196/16737)
Supplement: Multimedia Appendix 4 [file resprot_v9i8e16737_app4.pdf]

MEC-U
MEDICAL RESEARCH ETHICS
COMMITTEES UNITED

Medical research Ethics
Committees United

Postadres Postbus 2500
3430 EM Nieuwegein

Bezoekadressen Locatie Nieuwegein
Koekoekslaan 1 te Nieuwegein
030 609 3580

Locatie Eindhoven
Michelangelolaan 2 te Eindhoven
040 239 8607

E-mail info@mec-u.nl
Website www.mec-u.nl

Catharina Ziekenhuis
T.a.v. dr. M. A. Soliman Hamad, Research arts
Postbus 1350
5602 ZA EINDHOVEN

Kenmerk: V.127368/R17.051/ld/ld

Datum: 26-2-2018

Betreft: besluit NL62142.100.17
R17.051/E-coaching

Geachte heer Soliman Hamad,

Hierbij zend ik u het besluit van MEC-U inzake het protocol getiteld: **"The Effectiveness of Self-monitoring and Web-based Coaching in Promoting Physical Activity during Early Cardiac Rehabilitation: A Feasibility Study & A Randomized Controlled Trial"** (NL62142.100.17).

MEC-U verleent haar goedkeuring aan genoemd onderzoek. Voor de overwegingen bij het besluit verwijs ik u naar het bijgevoegde besluit.

MEC-U wijst u erop dat definitieve toestemming van de Raad van Bestuur nodig is voordat tot uitvoering van het onderzoek kan worden overgegaan.

Vriendelijk verzoekt de commissie u deze studie in te dienen bij de Lokale beoordelingscommissie ter verkrijging van een verklaring van geen bezwaar voor uitvoering van de studie in het Catharina Ziekenhuis. De indiening van een nieuwe studie loopt vanaf 8 mei 2017 digitaal via het programma Study Management. Meer informatie m.b.t. het indienen van de diverse documenten kunt u terugvinden op de website van Catharina Ziekenhuis Wetenschap, https://www.catharinaziekenhuis.nl/wetenschap/paginas/946-indieningsdocumenten.html

Ik vertrouw erop u hiermee voldoende te hebben geïnformeerd.

Met vriendelijke groet,

Mevrouw L. van Daal
secretaresse Medical research Ethics Committees United (MEC-U)

C.c. CCMO

BESLUIT
Primaire beoordeling

NL-nummer	NL62142.100.17	Registratienr.	R17.051
Titel onderzoek	The Effectiveness of Self-monitoring and Web-based Coaching in Promoting Physical Activity during Early Cardiac Rehabilitation: A Feasibility Study & A Randomized Controlled Trial		

Contactgegevens: Catharina Ziekenhuis, dr. M. A. Soliman Hamad, Postbus 1350, 5602 ZA Eindhoven
Verrichter: Catharina Ziekenhuis te Eindhoven

Besluit
De Medical research Ethics Committees United (MEC-U) heeft zich, op grond van artikel 2, tweede lid, sub a van de Wet medisch wetenschappelijk onderzoek met mensen (WMO), beraden over bovenstaand onderzoeksdossier.

De commissie oordeelt positief over het onderzoeksdossier uit te voeren in het volgende centrum
- Catharina Ziekenhuis te Eindhoven, hoofdonderzoeker drs. N.J. Verberkmoes

Documenten
Het oordeel is gebaseerd op de documenten die in bijlage 1 zijn vermeld.

Achtergrond
Op 18-07-2017 is het onderzoeksdossier ter beoordeling bij MEC-U ingediend. Het onderzoeksdossier is besproken in de vergadering(en) van 07-08-2017, 19-12-2017, 21-12-2017, 16-01-2017 en 08-02-2018 (zie bijlage 2 voor de aanwezige leden op 07-08-2017).
Op een vraagbrief en/of verzoek om aanvullende informatie vanuit MEC-U van 05-09-2017, 18-01-2018 en 08-02-2018 is door de indiener gereageerd op 30-10-2017, 31-01-2018 en 09-02-2018.

Overwegingen
MEC-U is van oordeel dat aan de voorwaarden in artikel 3, eerste lid, onder a t/m m, van de WMO is voldaan. De belangrijkste vragen en opmerkingen van de commissie betroffen:
- de productinformatie
- uitkomstvariabelen van de gerandomiseerde studie
- data storage
- de methodologie
- de statistische analyseparagraaf
- het verzoek om ontheffing proefpersonenverzekering
- de patiënteninformatiebrieven incl. toestemmingsformulieren

De belangrijkste argumenten van de commissie om over te gaan tot een positief besluit zijn dat de vragen genoegzaam zijn beantwoord en de documenten correct zijn aangepast.

De commissie heeft de in bijlage 1 vermelde onderzoeksverklaring bekeken. Zij heeft geconstateerd dat is voldaan aan de voorwaarden in artikel 3, onderdeel f van de WMO.

De commissie is van oordeel dat het onderzoeksprotocol in een toestemmingsprocedure voorziet die overeenstemt met artikel 6, eerste en derde lid, van de WMO.

De commissie is van mening dat is voldaan aan de voorwaarden in artikel 6, vijfde t/m negende lid, van de WMO. De proefpersonen worden op gepaste, volledige en begrijpelijke wijze schriftelijk over het onderzoek geïnformeerd en over de mogelijkheid om de toestemming te allen tijde in te trekken.

Deelnemende instellingen: St. Antonius Ziekenhuis te Utrecht/Nieuwegein, Catharina Ziekenhuis te Eindhoven, Diakonessenhuis te Utrecht/Zeist/Doorn, Meander Medisch Centrum te Amersfoort/Baarn en Onze Lieve Vrouwe Gasthuis te Amsterdam

Verzekeringen
De commissie is van oordeel dat ontheffing van de plicht tot het afsluiten van een WMO-proefpersonenverzekering kan worden verleend, op grond van artikel 7, zesde lid, van de WMO. De deelnemende proefpersonen lopen volgens de commissie geen of nauwelijks risico's.

De commissie heeft geconstateerd dat een aansprakelijkheidsverzekering is afgesloten zoals bepaald in artikel 7, negende lid, van de WMO.

Ten slotte wijst de MEC-U u op de voorwaarden en verplichtingen die in bijlage 3 zijn vermeld.

Met vriendelijke groet,

Mw. drs. M.S.E. van Poeijer, ambtelijk secretaris

Namens dr. B. van Ramshorst
voorzitter Medical research Ethics Committees United (MEC-U)

Nieuwegein, 26-2-2018

Beroepsprocedure
Tegen dit besluit kan een belanghebbende op grond van artikel 23 van de WMO binnen zes weken na de dag waarop het besluit is bekend gemaakt, administratief beroep instellen bij de Centrale Commissie Mensgebonden Onderzoek (CCMO). Het beroepschrift dient u te adresseren aan CCMO, Postbus 16302, 2500 BH Den Haag.

Deelnemende instellingen: St. Antonius Ziekenhuis te Utrecht/Nieuwegein, Catharina Ziekenhuis te Eindhoven,
Diakonessenhuis te Utrecht/Zeist/Doorn, Meander Medisch Centrum te Amersfoort/Baarn en Onze Lieve Vrouwe Gasthuis te Amsterdam

MEC-U
MEDICAL RESEARCH ETHICS
COMMITTEES UNITED

Bijlage 1

Documenten

A1	Aanbiedingsmail d.d.18-7-2017
A1	Vraagmail MEC-U d.d. 05-09-2017 en reactie indiener d.d. 30-10-2017
A1	Vraagmail MEC-U d.d. 18-01-2018 en reactie indiener d.d. 31-01-2018
A1	Vraagmail MEC-U d.d. 08-02-2018 en reactie indiener d.d. 09-02-2018
B1	ABR-formulier, versie 04 d.d. 08-02-2018
C1	Onderzoeksprotocol, versie 3.4 d.d. 08-02-2018
D6	Technical report Device safety d.d. 16-04-2014
D6	Declaration_CE_Axivity_KL.pdf.30102017
E1/E2	Patiënteninformatiebrief incl. toestemmingsverklaring(fase I), versie 1.4 d.d. 08-02-2018
E1/E2	Patiënteninformatiebrief incl. toestemmingsverklaring(fase II), versie 1.4 d.d. 08-02-2018
G1	Verklaring proefpersonenverzekering ondertekend d.d. 10-07-2016
G2	Bewijs dekking aansprakelijkheid van Stichting Catharina Ziekenhuis; polisnr. 624.455.403 van CentraMed d.d. januari 2017
H1	CV onafhankelijk deskundige dr. T.A. Simmers d.d. 19-06-2017
I2	Onderzoeksverklaring Catharina Ziekenhuis ondertekend d.d. 10-07-2017
I3	CV hoofdonderzoeker drs. N.J.E. Verberkmoes, ontvangen d.d. 02-08-2018
I4	GCP-WMO certificaat drs. N.J. Verberkmoes d.d. 22-05-2013

Deelnemende instellingen: St. Antonius Ziekenhuis te Utrecht/Nieuwegein, Catharina Ziekenhuis te Eindhoven,
Diakonessenhuis te Utrecht/Zeist/Doorn, Meander Medisch Centrum te Amersfoort/Baarn en Onze Lieve Vrouwe Gasthuis te Amsterdam

Bijlage 2

Samenstelling MEC-U
De volgende leden waren aanwezig tijdens de commissievergadering van 07-08-2017:

dr. B. van Ramshorst, voorzitter
dr. R.J.E. Grouls, klinisch farmacoloog / ziekenhuisapotheker
dr. E.J.F. Franssen, klinisch farmacoloog / ziekenhuisapotheker
dr. J.C. Kelder, klinisch epidemioloog
mw. ir. C. Kloeze
dr. P.J. Nickel, ethicus
mw. mr. Z.K. Ottovay, jurist
mw. A.E.H. Loth, patiëntenvertegenwoordiger

Deelnemende instellingen: St. Antonius Ziekenhuis te Utrecht/Nieuwegein, Catharina Ziekenhuis te Eindhoven,
Diakonessenhuis te Utrecht/Zeist/Doorn, Meander Medisch Centrum te Amersfoort/Baarn en Onze Lieve Vrouwe Gasthuis te Amsterdam

Bijlage 3

Voorwaarden en verplichtingen*

Geldigheid oordeel
Het positieve oordeel verliest zijn geldigheid als de inclusie van de eerste proefpersoon niet heeft plaatsgevonden binnen een jaar nadat dit besluit is genomen.

Amendementen
Amendementen dienen ter beoordeling aan MEC-U te worden voorgelegd.

Startdatum onderzoek
MEC-U dient op de hoogte te worden gesteld van de definitieve startdatum van het onderzoek. Dat is de datum waarop de inclusie van de eerste proefpersoon plaatsvindt.

Voortgangsrapportage
Eén jaar na datum van het oordeel, en ieder jaar daaropvolgend, dient MEC-U op de hoogte te worden gebracht van de voortgang van de studie middels het formulier Voortgangsrapportage.

Geldigheid verzekering
In het geval het verzekeringscertificaat tijdens de voortgang van het onderzoek zijn geldigheid verliest, dient aan MEC-U tijdig een afschrift van een nieuw geldig certificaat te worden toegestuurd.

Melding SAE's
SAE's dienen aan MEC-U te worden gemeld.

Melding (voortijdige) beëindiging en opschorting
(Voortijdige) beëindiging en opschorting van het onderzoek dient, met redenen omkleed, te worden gemeld aan MEC-U.

Eindrapportage
MEC-U dient op de hoogte te worden gebracht van de resultaten van het onderzoek middels een eindrapport.

Termijnen en overige uitleg ten aanzien van de indiening van de verschillende documenten aan MEC-U vindt u op de website van de CCMO.

Deelnemende instellingen: St. Antonius Ziekenhuis te Utrecht/Nieuwegein, Catharina Ziekenhuis te Eindhoven, Diakonessenhuis te Utrecht/Zeist/Doorn, Meander Medisch Centrum te Amersfoort/Baarn en Onze Lieve Vrouwe Gasthuis te Amsterdam
